# Bioinformatic Analysis of Pathogenic Missense Mutations of Activin Receptor Like Kinase 1 Ectodomain

**DOI:** 10.1371/journal.pone.0026431

**Published:** 2011-10-18

**Authors:** Claudia Scotti, Carla Olivieri, Laura Boeri, Cecilia Canzonieri, Federica Ornati, Elisabetta Buscarini, Fabio Pagella, Cesare Danesino

**Affiliations:** 1 Department of Experimental Medicine, Section of General Pathology, University of Pavia, Pavia, Italy; 2 Department of Human and Hereditary Pathology, Section of General Biology and Medical Genetics, University of Pavia, Pavia, Italy; 3 Department of Gastroenterology, Maggiore Hospital, Crema, Italy; 4 Department of Otorhinolaryngology, Fondazione IRCCS Policlinico San Matteo, ENT Unit, Pavia, Italy; 5 Medical Genetics, Fondazione IRCCS Policlinico San Matteo, Pavia, Italy; Naval Research Laboratory, United States of America

## Abstract

Activin A receptor, type II-like kinase 1 (also called ALK1), is a serine-threonine kinase predominantly expressed on endothelial cells surface. Mutations in its *ACVRL1* encoding gene (12q11-14) cause type 2 Hereditary Haemorrhagic Telangiectasia (HHT2), an autosomal dominant multisystem vascular dysplasia. The study of the structural effects of mutations is crucial to understand their pathogenic mechanism. However, while an X-ray structure of ALK1 intracellular domain has recently become available (PDB ID: 3MY0), structure determination of ALK1 ectodomain (ALK1_EC_) has been elusive so far. We here describe the building of a homology model for ALK1_EC_, followed by an extensive bioinformatic analysis, based on a set of 38 methods, of the effect of missense mutations at the sequence and structural level. ALK1_EC_ potential interaction mode with its ligand BMP9 was then predicted combining modelling and docking data. The calculated model of the ALK1_EC_ allowed mapping and a preliminary characterization of HHT2 associated mutations. Major structural changes and loss of stability of the protein were predicted for several mutations, while others were found to interfere mainly with binding to BMP9 or other interactors, like Endoglin (CD105), whose encoding *ENG* gene (9q34) mutations are known to cause type 1 HHT. This study gives a preliminary insight into the potential structure of ALK1_EC_ and into the structural effects of HHT2 associated mutations, which can be useful to predict the potential effect of each single mutation, to devise new biological experiments and to interpret the biological significance of new mutations, private mutations, or non-synonymous polymorphisms.

## Introduction

Activin A receptor, type II-like kinase 1 (also called ALK1, Uniprot entry P37023, protein family (pfam) 01064 of Activin types I and II receptor domains), is a serine-threonine kinase predominantly expressed on endothelial cells surface and it acts as a type I receptor for the Transforming Growth Factor-β/Bone Morphogenetic Protein (TGF-β/BMP superfamily of ligands. TGF-β/BMP signalling is induced when a dimeric ligand binds to the extracellular domain of two type I and two type II receptors [1]. This hexameric assembly permits interaction between the intracellular domains, with the constitutively active intracellular domain of type II receptor cross-phosphorylating the intracellular glycine-serine (GS) domain of type I receptor [2]. These receptor complexes can contain a type III receptor also termed a co-receptor (betaglycan [3], Endoglin [4] or RGM-a, b, c [5]) that modulates ligand affinity for its type I and type II receptors [6].

From the structural point of view, type I and type II receptors share a general fold resembling a class of neurotoxins known as three-finger toxins and hence called “three-finger toxin fold”. This fold is comprised from β-strands stabilised by disulphide bonds formed by conserved Cys residues. Three pairs of anti-parallel β-strands are curved to generate a concave surface. Despite the common architecture and the cluster of conserved Cys residues, very little sequence identity and no functional overlap exist between the two types of receptors.

BMPs consist of a Cys knot characterised by three pairs of highly conserved disulphide bonds in which one traverses through a ring formed by the other 2. This fold can be described as a hand with a concave palm side and two parallel β-sheet forming 4 fingers, with each β-strand being likened to a finger. Finger 2 leads to a helix “wrist” region. In the dimeric ligand the 4 fingers extend from the Cys core of the protein like butterfly wings. Binding of type I receptors occurs near the α-helix on the concave side at the junction between the two subunits [7], whereas binding to type II receptors happens on the convex side of the hand near the “fingertips” [8], [9].

ALK1 shares with other type I receptors a high degree of similarity in the GS domain, in the following serine-threonine kinase subdomains and in the short C-terminal tail [10], but the extracellular domain shows a peculiar aminoacidic sequence. ALK1 ligand has been elusive for a long time, but it has been recently demonstrated that BMP9 binds ALK1 in association with BMPRII or ActRIIA [11]–[14], inhibiting endothelial cell proliferation and migration. BMP9 triggers Smad1/5/8 phosphorylation trough ALK1/BMPRII in endothelial cells with an EC50 around 50 pg/ml (2 pM). This is a much higher affinity than that of other BMPs for their type I receptors: for example, BMP2 has an apparent Kd of 0.9 nM for ALK3 and 3.6 nM for ALK6 [14]. This feature suggests that the structural basis of ALK1 receptor binding might be different from other BMPs, which is further supported by the fact that, in contrast to all other type I receptors, ALK1 is missing residue F85, which was shown to be involved in the hydrophobic interactions between other BMPs and their type I receptors [15], [16]. Mutations in the components of this complex signalling system have been associated with diseases. Thus, Hereditary Hemorrhagic Telangiectasia (HHT) is an autosomal dominant multisystem vascular dysplasia characterized by mucocutaneous telangiectases and multiple arteriovenous malformations (AVMs) mainly in lung, liver and brain. Its HHT1 form is determined by mutations of type III receptor Endoglin (CD105), a homodimeric membrane glycoprotein coded by *ENG* (9q34) (OMIM*131195), while HHT2 depends on mutations of ALK1 coded by the *ACVRL1* gene (12q11-14) (OMIM*601284). The pathological basis of the associated vascular malformations is lack of intervening capillaries and results in direct connections between arteries and veins. HHT is a Rare Disease, with an incidence of 1 in 5-8000, likely underestimated. Penetrance is complete after the 4^th^ decade of life but a large inter and intra-familial variability in phenotype is observed. Moreover, a combined phenotype of HHT and Juvenile Polyposis is recognized as the JPHT syndrome, related to mutations in *MADH4* gene (18q21.1; OMIM*600993), coding for SMAD4, the common mediator of TGF-β/BMPs signalling, involved in transcriptional activation of as yet unidentified target genes [17].

To date, 329 different mutations have been reported for *ENG* (HHT1) and 272 for *ACVRL1* (HHT2) [18] with an uneven distribution of these mutation between North America/North Europe population (higher prevalence of *ENG* mutations) and Mediterranean populations (higher frequence of *ACVRL1* mutations). Our group reported an unusual distribution of mutations in Italy, with more than 30% of Patients carrying an *ACVRL1* mutation in exon 3, which codes for 98% of the extracellular domain [19]. An issue which has not yet been completely elucidated is whether mutated ALK1 is expressed or not. In fact, missense mutations and short in frame deletions and insertions often impair propensity of the affected polypeptide to fold to the functional conformation and/or decrease stability of the functional conformation [20]. Both effects lead to an increase of the proportion of mutant polypeptide present in non-functional conformations that are more susceptible to degradation or aggregation than the functional conformation [20]. Diseases with this kind of molecular pathogenesis are described as *conformational diseases*, and the interest for their pathogenic mechanism is not only academic: in fact, protein misfolding and aggregation may be an ideal therapeutic target for diseases caused by trafficking defects of misfolded secreted proteins. Recently, three mutants of ALK1_EC_ have been investigated and, though they barely reach the cell surface and do not bind BMP9, they are expressed in transfected cells [21], suggesting that alteration of structure determined by these mutations is likely responsible of the permanence of the protein in the cell and of the related pathogenic phenotype.

As reported in [22], several recent studies have applied one or a few bioinformatic methods to predict potentially deleterious effects of missense mutations in other diseases. However, the emerging trend is to utilise a more extensive set of prediction methods in order to attain more reliable results [22]. Many of them are based on protein sequence, but several are structure-based, as the latter are more reliable and provide more information. A model of the so far elusive three-dimensional structure of ALK1_EC_ could therefore provide insight into its molecular functions and be used to study the effect of disease-related mutations, like in [23]–[25]. In this work, we have built a homology model of ALK1_EC_ applying the most updated available methods, and we have investigated the predicted effects of HHT2-related missense mutations of ALK1_EC_ using multiple computational methods, including docking to the X-ray structure of BMP9. This approach allowed a preliminary characterization of ALK1_EC_ mutations, with prediction of their potential molecular pathogenic effect.

## Results and Discussion

In order to tackle mutation analysis by structure-based methods, we produced a homology model of ALK1_EC_. The first step involved identification of the three dimensional fold.

### Identification of the three dimensional fold

To create a model of ALK1_EC_, a BLAST search towards the PDB database was performed using residues 22–118 of ALK1_EC_ target sequence (Uniprot entry P37023). No significant similarities to other known structures were identified (minimum E value = 14). In contrast, a C-BLAST in the Conserved Domain Database found significant matches within protein family pfam01064, a domain class characterised by conservation of the CCX(4–5)CN motif. Though this observation and the absolute conservation of ten Cys residues throughout the type I receptors of the TGF-β superfamily [10] suggest a common fold for all its members, Cys-rich proteins are known to potentially generate alternative folding patterns, which would influence the choice of the correct template for homology modelling. In cases where template identification by sequence alignment fails or is uncertain, *ab initio* modelling methods are a possibility, but they are not yet performing sufficiently well according to the most recent 9th Community Wide Experiment on the Critical Assessment of Techniques for Protein Structure Prediction (CASP9, [26]). An alternative is identification of the correct three dimensional fold by threading methods, which allows assessment of the compatibility of the target sequence with the available protein folds based not only on sequence similarity, but also on structural considerations [27], [28]. In order to analyse this problem, ALK1_EC_ was submitted to the protein fold recognition metaserver Pcons [29]–[31], which submits the query sequence to multiple servers at the same time. All fold recognition servers found templates, with a similar, low sequence identity (21–23%). Particularly, FORTE, FUGUE, LFUGUE, LSP3 and SAM-T02 reported BMP receptor IA (PDB entries: 2h62C, 1es7B and 1rewC) as the best template, HHPRED2, LHHSEARCH15, LPROSPECT2, NFOLD and RPSBLAST reported BMP receptor IB (PDB entry: 3evsC), and LPPA-I and MUSTER, reported BMP receptor IA variant IA/IB (PDB entry: 2qjbD) as the best template. Only LSPARKS2 reported TGF-beta type 2 receptor (PDB entry: 2pjyC) as the best template and PHYRE the Urokinase plasminogen activator surface receptor, UPAR (PDB entry: 1ywhC). All of them are members of the “snake toxin-like” superfamily, “extracellular domain of cell surface receptors” family, according to SCOP database, and share the same fold. On the basis of these results, we can conclude that, despite the low sequence identity, comparative modelling can be considered an appropriate approach to predict the three-dimensional (3D) structure of ALK1_EC_.

### Prediction of an atomic model for ALK1_EC_

Availability of experimental 3D templates allowed us to create a 3D model of ALK1_EC_ by homology modelling, taking into account the difficulties encountered with low sequence identity (between 20 and 40%), a borderline case which has to be treated carefully [32]–[34]. Nevertheless, when proteins used for alignment and modelling belong to the same protein family in which the structure is well conserved, overall structural similarity can overcome the problem of low sequence identity [32]. Furthermore, model quality assessment and comparison of homology models generated by different algorithms is useful in order to identify problematic regions. In order to do this, top scoring models were selected among those obtained by each of the two metaservers Pcons [29]–[31] and Genesilico [35], the latter including modelling by multiple sequence alignment by the Frankenstein Monster approach [36], and I-Tasser [37] and RaptorX [38], the two servers giving the best homology modelling results for automated prediction of human targets using multiple-template threading in CASP9 [26], [39].

**Figure 1:**
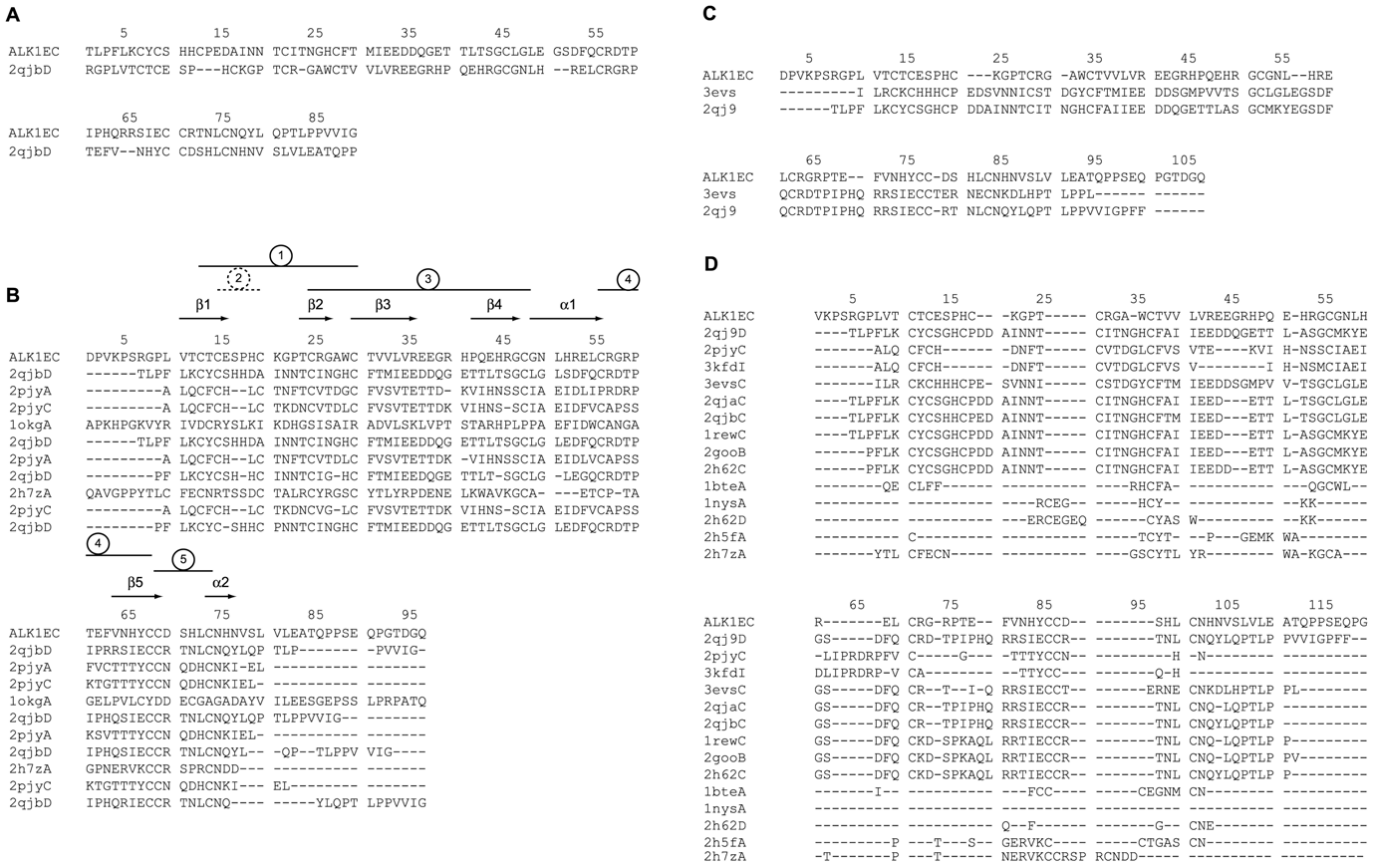
Structural alignments. Sequence alignment obtained using ALK1_EC_ by different structure prediction software types: (A) Pcons metaserver, (B) I-Tasser, (C) RaptorX, (D) Genesilico metaserver. In the alignment in (B) secondary structure elements are indicated. Disulphide bonds are numbered within circles. Number 2 is in a dashed circle to indicate that it would be destabilising in our ALK1_EC_ model. ALK1_EC_: ALK1 ectodomain sequence. Other sequence names are given as PDB IDs followed by chain name. 2qjb: Bone Morphogenetic Protein Receptor Type IA (ALK-3), 2pjy: TGF-β Receptor Type I (ALK-5); 1okg: 3-mercaptopyruvate sulfurtransferase from *Leishmania major*, 2h7z: irditoxin, 3evs: Bone Morphogenetic Protein Receptor Type IB (ALK-6), 2qj9: BMP-2 in complex with BMPR-IA variant B1, 3kfd: ternary complex of TGF-β1, 2qja: BMP-2 in complex with BMPR-IA variant B12, 1rew: complex of bone morphogenetic protein 2 and its type IA receptor, 2goo: BMP-2 bound to BMPR-Ia ectodomain and ActRII ectodomain, 2h62: Bone Morphogenetic Protein Receptor Type IA (ALK-3), 1bte: extracellular domain of the type II activin receptor, 1nys: Activin A bound to ActRIIB P41 ectodomain, 2h5f: denmotoxin.

**Figure 2:**
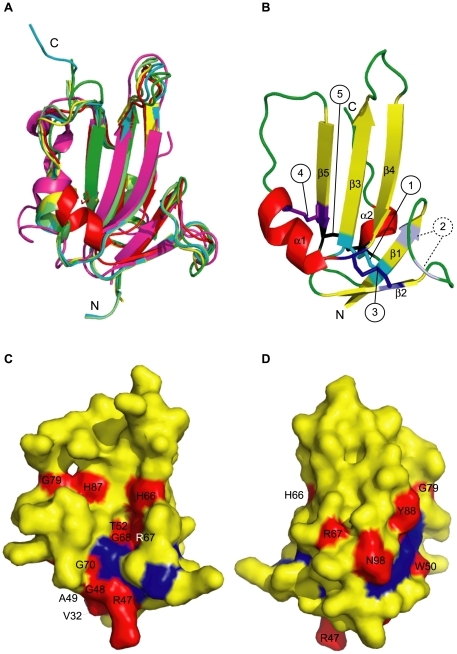
ALK1_EC_ model. (A) Cartoon representation of the most recurrent templates superposed onto the final ALK1_EC_ model. Red: final ALK1_EC_ model; cyan: 2qjbd; magenta: 2pjyc; green: 3evsc; yellow: 2h62c; light green: 2qj9c. (B) Cartoon representation of secondary structure elements of the final ALK1_EC_ model. The typical three-finger toxin structure is visible. Yellow: β-strands, red: α-helices, green: loops. Disulphide bonds are displayed as sticks and numbered in circles according to Fig. 1B. Dashed circle indicates a lacking disulphide bond: conserved in the templates, it would introduce strain in the model. (C) and (D) Surface mapping of HHT2-related mutation sites on ALK1_EC_ model (yellow). Views of the concave (C) and convex (D) surface are displayed. In red: non-Cys mutational sites, labelled according to residue number. In blue (unlabelled for clarity): Cys residues. Non-Cys mutational sites are mainly clustered in the lower two-thirds of the molecule. Figures were prepared with Pymol [106].

Pcons metaserver top scoring model (Pcons score: 0.352) was the one based on the single target-template alignment obtained by LPPA-I fold recognition server using structure 2qjbD as a template (Fig. 1A) and was better than those generated by multiple sequence alignments by the same metaserver, as evaluated by PconsM (data not shown). Sequence alignments used by I-Tasser, RaptorX and Genesilico are shown in Fig. 1B, 1C and 1D, respectively. Global Qmean scores of each generated models ranged from 0.36 (I-Tasser) through 0.49 (Pcons) and 0.56 (RaptorX) to 0.57 (Genesilico), indicating a significant variability of model quality. However, superposition of the four models demonstrated that they shared a virtually identical general fold, with a maximum RMSD of 1.96 Å between Cα traces of Pcons and I-Tasser generated models. Analysis of Qmean local scores for each model by superposition of Qmean server-generated PDB files (Fig. S1A) indicated that in all the models strands β1, β2 and β3 were consistently reliable. β4 and β5 strands had slightly higher local scores, which became even higher in the remaining part of the polypeptide, especially loops. As a comparison, however, local Qmean scores for structures available in the same protein family, like 2h62C and 3evsC, were only slightly higher than those of the generated ALK1_EC_ models. This supported the fact that the four models had a significant reliability. In order to further improve the results and obtain a single final model, MODELLER was used [40], using the four server-generated models as templates. In this step, a α helix for residues 70–76, a secondary structure element recognised by PSIPRED [41] and not present in the starting structures, was also imposed. The resulting model was then evaluated by Qmean and showed a significant increase in the global Qmean score (0.603, Fig S1B). As a further assessment of model quality, structural variability within family pfam 01064, measured with an all-versus-all comparison through the ProCKSI server (www.procksi.net), gave an average TM-score of 0.62±0.20, while the same parameter measured for the final model versus its structural templates was 0.71±0.070 (t-test: P = 0.33)”.Fig. 2A depicts the model of ALK1_EC_ (in red) overlapped on the most recurrent templates used in the modelling procedure: Bone Morphogenetic Protein Receptor Type IA (ALK-3, 2qjb, cyan), TGF-β Receptor Type I (ALK-5, 2pjy, magenta), Bone Morphogenetic Protein Receptor Type IB (ALK-6, 3evsc, green); Bone Morphogenetic Protein Receptor Type IA (ALK-3, 2h62, yellow); BMP-2 in complex with BMPR-IA variant B1 (2qj9: light green). Assessment of ALK1_EC_ model by RAMPAGE for stereochemical quality [42] showed 92.9% of residues in favoured regions, 4.3% of residues in allowed regions, and 2.9% residues in disallowed regions. The two residues in disallowed regions (S38 and T82) belong to the first and fourth loops and further optimisation to improve their phi psi angles led to a reduction of Qmean score. Model validation was also performed by ProSA-web [43], which gave a very good Z-score of -5.45 and showed that the plot of the local model quality (energies as a function of amino acid positions) was consistently negative for all of them, confirming the absence of problematic parts (Table 1) [44]. VERIFY3D [45], ProQ LG [46], and ProQ MaxSub [46] scores were not too far or even better than those of the template crystal structures (Table 1). Threading energy was also comparable to the template structures.

**Table 1:** Quality assessment of representative template structures and of ALK1_EC_ model.

Molecule (PDB ID)	Qmean [76]	Verify 3D [45]	ProQ LG [46]	ProQ MaxSub [46]
2h62C	0.73	22.13	2.051	0.306
2qjbD	0.72	24.02	1.072	0.173
3evsC	0.74	25.11	1.370	0.220
2qj9D	0.77	24.90	1.208	0.184
2pjyA	0.71	17.33	1.228	0.209
ALK1_EC_	0.60	19.90	1.355	0.231

In general, the results shown in Table 1 and the superposition of Fig. 2A indicate that, though the final model is not as good as the crystallographically determined reference structures, as expected because of the low sequence identity to the templates, it was anyway sufficiently good to derive some functional inference. The model was deposited in the Protein Model Database (PMDB) with code PM0077425.

### Model description

The final model included residues 31–107 (Fig. 2B). The general shape of ALK1_EC_ model is of a cupped left hand, with the thenar eminence corresponding to α-helix 1, including residues 70–77, and the thumb to the loop formed by residues 78–84. The core region of the model exhibits the characteristic three-finger toxin fold (Fig. 1 and Fig. 2B), with strand β1 including residues V32-E37, β2 residues T45-G48, β3 residues C51-R57, β4 residues P63-G68 and β5 residues V85-C90. Strands 1 and 2, and 2 and 3 are joined by a short loop, while strands 4 and 5 by a long, partially unstructured loop, including residues N71-V85 and a short α-helix (N95 and H98). Only four (C34-C51, C46-C69, C90-C95) of the expected five disulphide bonds were predicted: none of the four templates used in the Modeller modelling step included the bond one between C36 and C41, which is instead present in the crystal structures of all the other molecules of the same class. This might depend on the fact that the loop formed by residues E37-H40 includes 4 residues, among which a Pro. All the other members of the family have 3 or 5 residues loops and do not include Pro. These two specific elements (unique loop length and Pro presence) could explain why forcing Modeller to include a disulphide bond in the final model lead to a worsening of the Qmean score, especially because of alteration of torsion angles. This disulphide bond, therefore, was not included in the final model.

### Mutation analysis

In general, models with low sequence identity to the template like the one of ALK1_EC_ cannot be used for detailed predictions of the effects of mutations. Nevertheless, thanks to the low deviation of the Cα atoms positions with respect to templates, the results of our analysis can be used to put forward new hypotheses and may be helpful in guiding the design of further experimental research.

At the moment of writing, 32 HHT2-related positions for missense mutations have been described for ALK1_EC_ (HHT mutation database, [18]). Mutational sites P26, P30 and S110 were not included in our systematic bioinformatic analysis, as these residues are not part of our structural model.

**Figure 3:**
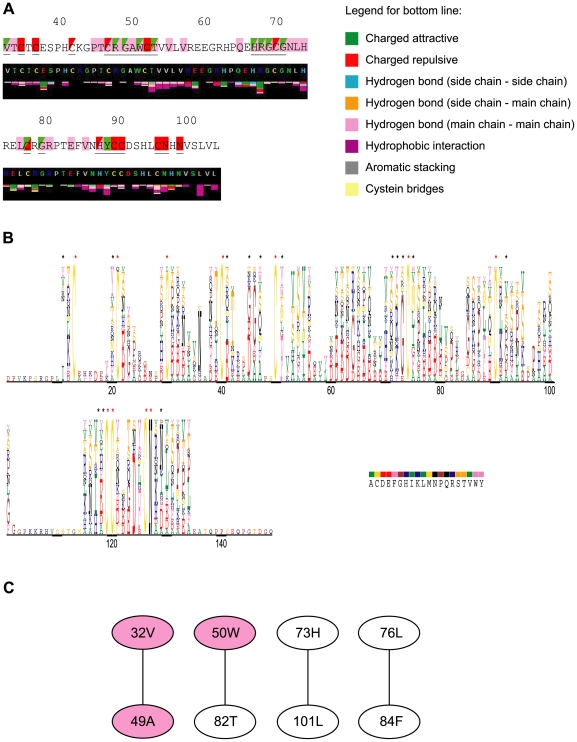
Bioinformatic analysis of HHT2 related ALK1_EC_ missense mutations. (A) Comparison of HHT2 related mutations with residues contacts. Top line: missense mutations are underlined in the sequence of ALK1_EC_. Red and green: mutations with a higher and lower impact, respectively, on protein folding according to our bioinformatic analysis (see also Table 2). Pink: residues involved in interactions with BMP9. Triangles are used to allow visualization of two colours when needed. Bottom line: Sting [64] output for contact analysis. Residue colour legend: grey: small and hydrophobic; green: polar; red: negatively charged; blue: positively charged; yellow: disulphide forming cysteines. (B) MultiDisp [52] output of the sequence alignment of ALK1_EC_ with its homologues. The height of the character is proportional to the frequency of the amino acid in that position. Similar colours are used for residues with similar physicochemical properties. Red asterisks indicate absolutely conserved residues, black ones other mutational residues. (C) Covarying residues determined with the program ProCon (p-value 0.001). Mutational sites are highlighted pink. Figures were prepared with Pymol [106].

**Figure 4:**
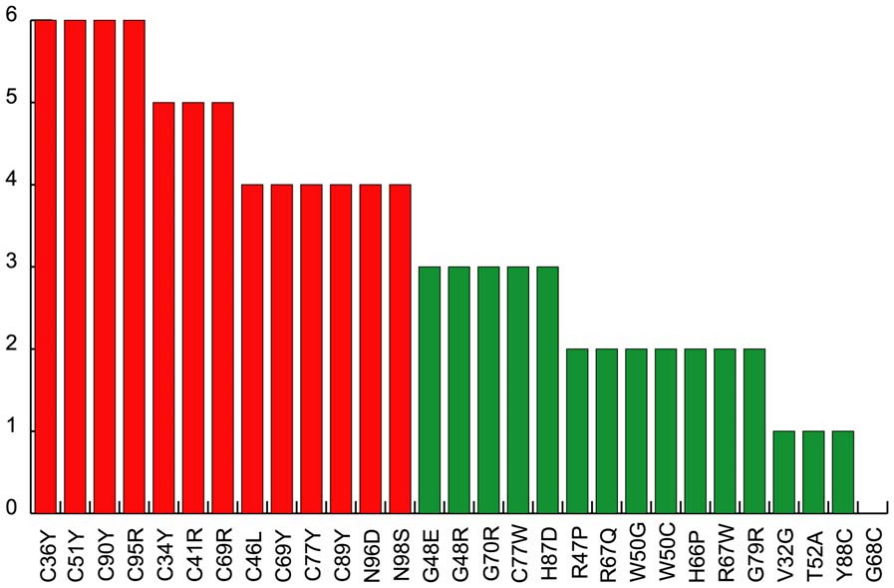
Graphical representation of the effects of mutations. The chart summarizes the effect of mutations on ALK1_EC_ protein structure. Red and green bars: mutations with a high and low, respectively, destabilizing effects on protein structure.

Mutation positions are underlined in Fig. 3A and were mapped on the calculated model of ALK1_EC_ domain in Fig. 2C and Fig. 2D, where mutational sites not involving and involving Cys residues are highlighted in red and blue, respectively. A visual analysis of the mutation positions allowed to observe that they are located in only two-thirds of the domain body, with the tip of the fingers completely untouched. They involve residues located both on the concave and convex surface and the wrist of the hand, affecting all Cys and some non-Cys residues. In order to better characterize the mutations, an extensive bioinformatic analysis was performed, according to Thusberg and Vihinen [22], with modifications described in the Material and Methods section. Results are summarised in Table 2 and Fig. 4, and discussed below.

**Table 2:** Summary of the effects of ALK1_EC_ mutations.

Mutation	Conserved residues	Electrostatic potential	Conformational	Contacts and stability	Disorder	Aggregation	Functional mutations	Contacts with BMP9
**V32G**	-	-	-	-	x	-	-	x
**C34Y**	x	-	x	x	x	-	x	-
**C36Y**	x	x	x	x	x	-	x	-
**C41R**	x	x	-	x	x	-	x	-
**C46L**	x	-	-	x	x	-	x	-
**R47P**	-	x	-	x	x	-	-	x
**G48E**	-	x	-	x	x	-	-	x
**G48R**	-	x	-	x	x	-	-	x
**W50G**	-	-	-	x	x	-	-	-
**W50C**	-	-	-	x	x	-	-	-
**C51Y**	x	x	x	x	x	-	x	-
**T52A**	-	-	-	x	-	-	-	x
**H66P**	-	x	-	x	-	-	-	x
**R67W**	-	x	-	x	-	-	-	x
**R67Q**	-	-	-	x	x	-	-	x
**G68C**	-	-	-	-	-	-	-	x
**C69R**	x	x	-	x	x	-	x	-
**C69Y**	x	-	-	x	x	-	x	-
**G70R**	-	x	x	x	-	-	-	x
**C77W**	x	-	-	x	-	-	x	-
**C77Y**	x	-	-	x	x	-	x	-
**G79R**	-	x	-	-	x	-	-	x
**H87D**	-	x	-	x	-	x	-	x
**Y88C**	-	-	-	x	-	-	-	-
**C89Y**	x	-	-	x	-	x	x	-
**C90Y**	x	-	x	x	x	x	x	-
**C95R**	x	x	x	x	x	-	x	-
**N96D**	x	-	x	x	-	-	x	-
**N98S**	-	x	-	x	-	x	x	-

#### Sequence conservation

Pathogenic mutations typically involve conserved positions within a protein family, as these involve residues essential for the structure and/or the function of a protein [22], [47]–[49]. In fact, the probability that a random mutation can cause a genetic disease has been shown to increase with an increase in the degree of site conservation [50]. The nature of amino acid substitutions in invariant sites will condition the effect on protein structure, while variable positions can be analysed for residues that can be exchanged without detrimental effects. Pfam [51] multiple sequence alignment for ALK1_EC_ confirms that there are 11 invariant positions (the 10 Cys and N96), all of which modified by one or several disease-related mutations. Fig. 3B shows the chemical nature of amino acids in MultiDisp output [52], with asterisks above mutated positions: red for absolutetly conserved positions and black asterisks above the others. Several missense mutations involving the 10 absolutely conserved cysteine residues have been identified so far: C34Y, C36Y, C41R, C46L, C51Y, C69R, C69Y, C77Y, C77W, C89Y, C90Y, C95R. Another absolutely conserved position is N96, which is mutated to D in a disease phenotype. In fact, N96 belongs to the Pfam characterising motif CCX(4–5)CN. Consurf [53] analysis of all Pfam multiple sequence alignments recognises all Cys residues as structurally important, and N96 as a functionally important residue.

Fifteen mutations significantly alter the physicochemical properties of wild-type amino acids as predicted by ProCon [54]. Two hydrophobic residues (V32 and W50) are replaced by Gly, an amino acid with a conformational role and with much a smaller size. Three Gly residues (G48, G68 and G70) are changed either into charged residues (Glu or Arg) or into a hydrophobic Cys. The former mutation might not be, however, very disruptive, as Glu is found in this position in some members of the Pfam group analysed by all the different alignment algorithms. The positively charged H66, R67 and H87 are mutated, respectively, into a conformationally important residue (Pro), which is likely to interrupt the continuity of the β-strand, into a hydrophobic residue (Trp) and into an oppositely charged residue (Asp). A Gln in position 67, on the other hand, might be more easily tolerated, as it is found in homologues by all MSA generators used. It is expected, therefore, that mutations G48E and R67Q will lead to a less disruptive action from the structural point of view.

There are four pairs of covarying residues (Fig. 3C), with 3 amino acids mutated in HHT2. In summary, our analysis shows that pathogenic mutations are located not only in absolutely conserved positions, but also in residues with a low level of evolutionary conservation.

#### Structural disorder and β-aggregation

Disorder and aggregation propensity of a protein can be increased by missense mutations, leading to loss of a regular secondary structure fold. These mechanisms have been recognised to be involved in Alzheimer's [55], Huntington's diseases [56], amyloidosis [57] and even aging [58]. At least three of the seven methods used predicted six mutations as able to increase disorder, and at least two of the four methods used predicted four of them as potentially able to influence aggregation.

#### Stability

The most frequent effect of missense mutations is alteration of protein folding and decreased stability [20]. Stability centres were predicted by Scide [59] and Scpred [60] and stabilizing residues by Sride [61]. Only mutated residue W50 was found to belong to the first group, while no residues were found to exert an essential stabilizing effect. However, when these results were considered together with those obtained with the 8 softwares used to test changes in stability upon mutations, all amino acid replacements were predicted to be destabilising by at least 4 methods, or, in the case of mutations C41R, W50G, C51Y, C69R, C69Y, H87D, C89Y, C95R and N98S , by at least 6 methods.

#### Structural mutations

**Figure 5:**
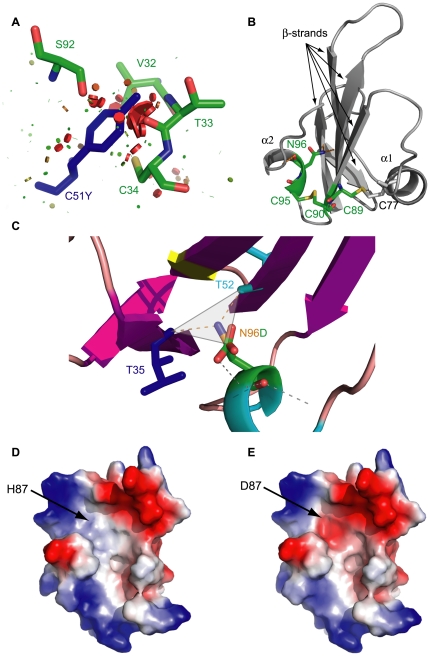
Structural effect of mutations. (A) Mutation C51Y causes major clashes with neighbouring residues. (B) Structural role of the highly conserved CCX(4-5)CN motif. The central β-strands is connected with α1-helix by disulphide bond C77-C89 and with α2-helix by disulphide bond C90-C95 combined with the hydrogen bonds formed by the N side chain atom of N96. Mutations affecting this motif have a high structural impact. (C) N96D mutation removes the N96 side chain N atom and its replacement by the C atom of D96 alters the hydrogen bond network. (D) and (E) show the effect of mutation H87D on electrostatic surface potential (from neutral to negative).

Amino acid replacement can determine major structural alterations, mainly determined by the physico-chemical properties of the new residue. Analysis of the fitting of each new side chain was performed using structural models generated by FoldX, which adopts a probability-based rotamer library, while exploring alternative conformations of the surrounding side chains. For each mutant, van der Waals clashes were compared with the corresponding wild-type structures using the corresponding energy values. Wild-type ALK1_EC_ had a van der Waals energy of –0.06 kcal/mole, while 12 out of the 28 mutants analysed showed higher values, comprised between 1.8 kcal/mole for C89Y and 26.14 kcal/mole for C51Y, indicating a strong local perturbation to the structure. Each mutant was analysed by Pymol and clashes visualised by a specific python function written by Thomas Holder (show_bumps.py, personal communication). Seven of the 12 mutants with high energy showed a clear bad fitting, with potential detrimental effects on folding (Table 1, “Conformational” column). A representative case of these mutations is illustrated in Fig. 5A.

Mutations introducing proline residues, like R47P and H66P are located at the base and in the concave surface of the “hand”, respectively. The former is likely to determine a change in the conformation of the β-strand including residues 45–48, while the latter affects one of the two symmetric His residues (H66 and H87) located in the middle of β-strand 2.

#### Mutations in contacts maintaining stability

Accessible surface area measurements performed by Areaimol [62], [63] indicate that ALK1_EC_ includes, despite its small size and its mainly β secondary structure, ten completely buried residues. Out of these, nine are mutational sites (C34, C36, C46, C51, T52, G68, G70, C95, N96), which is consistent with the notion that buried residues are typically involved in the formation of core interactions crucial for protein stability and that the probability of a mutation to be pathogenic is inversely proportional to the solvent accessible surface of the wild-type residue [50].

Residues T52, R67, Y88, C95 and N96 form a high number of bonds with neighbouring aminoacids, as displayed by Sting analysis [64] (Fig. 3A). All the mutations involving these residues involve a reorganization of the bond network, and could thus contribute to alteration of protein stability. However, it must be taken into account that, when interactions in the wild-type protein are mediated by main chain – main chain contacts, they are less susceptible to be broken by missense mutations. This happens, for example, in Y88C.

Mutations affecting Cys involved in highly conserved disulphide bonds are known to strongly alter protein stability and folding. This is consistent with the results of our bioinformatic analysis (Table 2) and with biological data reported by Ricard et al. [21]. A specific feature of the Pfam family ALK1 belongs to is the presence of the CCX(4–5)CN motif, with C89, C90, C95 and N96 the corresponding residues involved in ALK1_EC_, respectively. Their crucial structural role depends on the fact that, in all the members of the Pfam protein family whose structures are known, the first Cys residue of the motif (C89) forms a disulphide bond with the Cys comprised in the α1 helix (C77 in ALK1_EC_), while the second Cys residue (C90) forms a disulphide bond with the third Cys of the motif (C95), thus placing the following Asn residue (N96) in a favourable position to interact with the N terminal β1 strand of the domain (Fig. 5B). N96 is thus the pivotal residue around which the entire structure is folded. Ideally, its ND2 atom is at the centre of a triangular structure, wherefrom three bonds depart. One is the covalent bond with the CG atom of the same residue, and the other two the hydrogen bonds formed with T35 and T52, sewing the N terminal β1-strand with the β3 (middle finger) and the C terminal strands, with the latter generating the convex surface of the ectodomain (Fig. 5C). In the N96D mutant (Fig. 5D) all these bonds are lost, with a likely alteration of protein fold and of the proper orientation of sugars potentially linked to N98. Biological data supporting the results of our analysis are given in [21], where mutations C51Y, C77W and N96D have been studied into details, and demonstrated to allow expression of the corresponding mutated protein, though imparing its exposure on the cell surface. No measurements concerning folding status of mutant proteins or subcellular localization was performed in this work. However, the fact that mutants are detectable in western blot [21] suggests that they cannot reach the cell surface despite correct protein synthesis. In fact, misfolding in mutated proteins has been described as a major cause of impaired surface expression for the neural cell adhesion molecule L1 [65], with other examples represented by the cystic fibrosis transmembrane conductance regulator (CFTR) mutants [66], [67], most forms of α1-anti-trypsin deficiency [68], or Charcot-Marie-Tooth disease caused by missense mutations in the connexin-32 gene [69] (a review is in [20]). In all these cases, the mutated protein is misfolded, it is recognized as abnormal and, hence, retained in the endoplasmic reticulum where it is degraded by the “quality control” machinery. Correct folding and oligomerization of newly synthesized membrane and secretory proteins are prerequisites for export from the endoplasmic reticulum. Mutant and misfolded polypeptides or unassembled subunits of oligomeric proteins are retained in this organelle and ultimately degraded (reviewed in [70]). The mechanism by which mutants are recognised and disposed of by this apparatus are not well understood. *In vitro* folding studies would be useful in this respect for ALK1_EC_, as several mutations might interfere with correct folding of the polypeptide and decrease its stability [71].

Since only main chain atoms of the two residues hydrogen bonded to N96 are involved in structure stabilization, their mutations would be considered unlikely to determine strong fold alterations. T35 is not a mutational site. On the other hand, mutation T52A in the mutated FoldX-generated model alters the bond network involving N96 in a way totally similar to mutation N96D. Moreover, residue T52 is involved in interactions with H73 through its side chain OH, useful to optimize the orientation of the α1 helix against the concave surface of the hand. In this case, replacement of the side chain of T52 with an Ala would lead to loss of this potential interaction. Alternatively, T52 might also be involved in ligand binding, a hypothesis that we tested by docking simulation (see below).

R67, beyond binding E65 on the same β-strand, establishes hydrogen bonds with E37 from the N-terminal strand and with H97 from the helix of the convex surface. Therefore, it might have a role complementary to N96. Replacement of R67 by Trp disrupts these bonds, potentially jeopardizing the stability of the convex surface of ALK1_EC_.

#### Effects on electrostatic potential

Fourteen out of the 29 known ALK1_EC_ mutations determine alterations in the electrostatic potential: a hydrophobic to negative shift is caused by mutation G48E and H87D (Fig. 5E), and the reverse by mutations C36Y and H66P. A positive to hydrophobic shift is introduced by mutation R47P, while an increase in the negative surface extent is determined by C51Y and R67W. The most frequent alteration consists in the hydrophobic to positive shift induced by mutations C41R, G48R, C69R, G79R, and C95R. Interestingly, G48E and G48R induce an important alteration of surface charge distribution, introducing a large negative and positive patch, respectively, on a hydrophobic area of the convex surface of ALK1_EC_. All these alterations might have an effect on the interactions with BMP9, endoglin, and other potential ligands, but it is worthwhile noting that also surface charges are relevant in maintaining protein stability.

#### Surface mutations

According to our model, non-Cys mutations involve both the concave and the convex surfaces of ALK1_EC_. Particularly, mutational sites R47, T52, H66, R67, G68, G70, G79, H87 are located on the former. Of these, H66 and H87 are symmetrically located at the sides of a vertical hydrophobic groove whose floor is formed by residues V53, V54, L55, V56, F84. Five of the mutated non-Cys positions, occupied mainly by hydrophobic residues in the wild-type (V32, G48, A49, W50, G70, Y88), cluster instead on the convex surface of ALK1_EC_ suggesting that they might be located in a critical region for protein-protein interactions. In fact, changes introduced by the described missense mutations determine important alterations in size and charge (V32G, G48R, G48E, W50G, W50C, G70R, Y88C, C89Y), which would significantly alter the conformation of this surface region.

#### Functional mutations

ALK1_EC_ conserved residues have also a functional role, related to their being essential for a correct protein folding. Thus, all mutations affecting Cys residues and residues N96 and N98 belong to this class.

#### Pathogenic mutations

All the ALK1_EC_ mutations described here are known to be pathogenic. SIFT predicted all of them to be potentially damaging except for Y88C. Pmut predicted only T52A and N96D as tolerated instead of deleterious. Polyphen and PhDSNP considered 6 and 13 mutations, respectively, as tolerated.

#### Subsets of mutations

A summary of the data presented in Table 1 is illustrated in Fig. 4, where bars represent the score (total number of crosses) obtained in our analysis by each mutation. The chart suggested that it might be possible to hypothesise a preliminary classification of ALK1_EC_ missense mutations: mutations probably leading to protein misfolding and impairment of ALK1 surface expression by protein aggregation or by lack of binding of key components of the secretory pathway (Table 1 and on the left in Fig. 4), and non-destabilizing mutations, which might allow a significant or normal cell surface expression of ALK1, mainly exerting their pathogenic effect by interference with BMP9 or co-receptor binding (Table 1 and on the right in Fig. 4). Discrimination between the two classes by a clear-cut threshold was however difficult at this stage. We then decided to test our model in a docking simulation to determine which mutational residues would be expected to reside in interacting surfaces.

### Prediction of the interaction mode of ALK1_EC_ with BMP9

**Figure 6:**
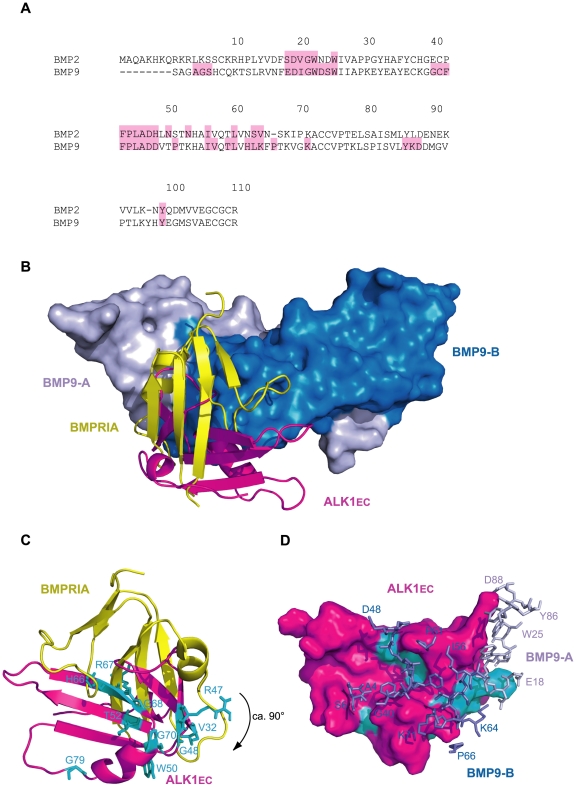
ALK1_EC_-BMP9 docking simulation. (A) Sequence alignment of BMP2 from PDB structure 1es7 and BMP9 from PDB structure 1zkz. Sequence numbering according to BMP9. Residues involved in type I receptor binding are shaded in pink. For BMP9, interface residues were calculated with PISA [108]. (B) Superposition of ALK1_EC_/BMP9 complex, as calculated by ClusPro onto BMP2/BMPRIA (PDB ID: 1es7) by BMPs structural alignment. In two shades of blue: surface of BMP9 subunits A and B (BMP2 not shown for clarity). In yellow and magenta: cartoon representation of BMPRIA and ALK1_EC_, respectively. (C) BMPRIA and ALK1_EC_ from (B) are visualised from the interface surface. The latter is rotated by about 90° with respect to the former. Mutational positions of ALK1_EC_ contacting BMP9 are shown as cyan sticks. (C) ALK1_EC_-BMP9 complex simulation: surface representation of ALK1_EC_ (magenta) and stick representation of BMP9 segments involved in binding (dark blue: subunit B, light blue: subunit A). Cyan: non-Cys mutational sites from (C), in contact with BMP9.

We performed a docking simulation with ClusPro 2.0 [72] between ALK1_EC_ and the structure of the dimeric form of BMP9 [PDB: 1ZKZ]. Though to be considered with great caution for the errors intrinsic to the ALK1_EC_ model used, the top scoring model of the complex (Protein Model Database code PM0077426) suggests a binding mode strikingly similar to the one between BMP2 (31% identity to BMP9, Fig. 6A) and BMPR-IA complex (PDB ID: 1es7, Fig. 6B). Interaction with BMP9 occurs at the composite interface formed by the two ligand monomers, exactly the same kind of binding strategy displayed by other receptors of the same class (e.g. PDB IDs: 1es7, 2h62; Fig. 6B). This result can be considered an important, indirect confirmation of the reliability of our ALK1_EC_ model. Moreover, in the complex with BMP9 ALK1_EC_ is oriented with a rotation by 90° with respect to BMPR-IA (Fig. 6B). This is in line with the view that, in these families of molecules, variable structural strategies for complex formation provide the specificity of interaction and hence of final signalling cascade. BMP9 residues involved in binding have a striking similarity with those recruited at the interface in the BMP2-BMPR-IA complex (PDB ID: 1es7, Fig. 6A, pink). Extra-residues are also involved in the case of ALK1_EC_-BMP9 interface (Fig. 6A, pink), indicating a more extensive interaction surface, which could explain the very high affinity measured in *in vitro* experiments for this complex [14].

Some important observations can be made. First of all, in both complexes ligand-receptor interactions occur mainly through hydrophobic patches. The concave surface of BMPR-IA is largely hydrophobic due to residues F60, G76, M78 and I99 and the disulphide bridge between C77 and C53. It is interesting to note that none of these residues is conserved in ALK1_EC_, except for G76 (G68 in ALK1_EC_). However, a wide hydrophobic surface area is present in the central part of the concave surface thanks to residues V54, V56, F84, V85 and L103 (Fig. 5C). In BMPR-IA, the hydrophobic concave surface is filled by residues from the pre-helix loop of BMP2, particularly F49, P50 and A52. A key feature of BMPR-IA binding is residue F85, which sticks out of the receptor helix α1 and fits, with a knob-into-hole packing, into a hydrophobic pocket of the ligand [7]. All of the pocket forming residues of BMP2 are invariant or highly conserved within the TGF-β superfamily, including BMP9 [73]. In fact, a highly hydrophobic residue corresponding to F85 of BMPR-IA is found in all type I receptors and has been proposed as a key feature of the type I receptor binding site [7]. However, in ALK1_EC_ the critical residue F85 is replaced by E75, clearly unfit to bind the hydrophobic binding pocket. This sequence feature of ALK1_EC_ suggested *per se* that binding of BMP9 was likely to occur through interactions different from those observed in the BMP2/BMPR-IA complex. In fact, the ca. 90° rotation (Fig. 6B and 6C) perfectly sorts out this charge problem, moving E75 completely outside the binding interface.

It is intriguing that the top scoring complex model showed an interaction interface with BMP9 including 10 out of the 14 non-Cys missense mutational positions for ALK1_EC_. They include, for the interface between ALK1_EC_ and BMP9 monomer A, residue R47, and for monomer B: V32, R47, G48, T52, H66, R67, G68, G70, G79 and H87. All these 10 mutational hotspots include residues whose replacement is not highly destabilizing according to our analysis (Table 2, green bars in Fig. 4, Fig. 6). Two residues, Y88 and W50, do not seem to affect ALK1_EC_ structure or its binding to BMP9. For its localization on the convex surface of ALK1_EC_, Y88 might be involved in its interactions with another partner, like, for example, a co-receptor. A similar line of reasoning can be applied to residue W50, targeted by pathogenic mutations W50G and W50C, which do not exert a crucial structural modification role and are not involved in interactions with the ligand.

The results of this simulation were also a further, indirect confirmation that the group of mutations represented with red bars in Fig. 4 affect residues which are more likely to have a structural role.

### Conclusions

HHT2-associated missense mutations detected in ALK1 result in a clinically relevant phenotype due to receptor functional impairment. Thus, they offer an invaluable source of information for protein genotype-phenotype correlation, as they can demonstrate the importance of wild-type residues located in mutational spots in determining the correct molecular conformation and/or in mediating interactions at the ligand-receptor interface. The rationale of our work relies on the fact that the study of the molecular basis of diseases by experimental methods is difficult and time-consuming, and prediction of the structural effects of pathogenic mutations may optimise the design, and reduce the number, of targeted biological experiments. The multiple combined bioinformatic methods, which we have applied to HHT2-related ALK1_EC_ pathogenic mutations required generation of a three-dimensional homology model of ALK1_EC_, the first good-quality model of ALK1 receptor ectodomain proposed so far. Consistency between independent predictions, particularly of HHT2 related missense mutation effects and docking simulation, is quite striking and suggests that a preliminary classification of the 29 ALK1_EC_ missense mutations here analysed would include three groups, affecting: residues mainly involved in protein structure stabilization (14 out of 29), residues mainly involved in interaction with BMP9 (12 out of 29), and, finally, residues likely to be involved in interactions with other partners, probably coreceptors (3 out of 29). These data lead to hypothesise that the similar clinical phenotypes of HHT2 might actually depend on alteration of at least three different molecular pathways or mechanisms: protein misfolding (thus configuring a conformational disease), ligand binding disruption or interference with co-receptor binding.

It is important to consider that each bioinformatic method investigates a specific aspect of the sequence or structure under consideration and implementing a considerable number of methods is a common strategy to integrate their strengths and overcome their weaknesses. Metaservers apply this philosophy on a wide scale for homology modelling and integrate statistical methods to assess the results. As a maximum number of methods is tested per each query sequence, integration of a system to automatically assess the results from different metaservers could be very useful to speed up and improve homology modelling. Protein threading itself is being the object of much improvement effort, especially to optimize alignments and energy functions [74], while assessments methods could be improved in order to better identify regions that can be trusted, with unreliable parts piped automatically to systems to improve them.

In the bioinformatic determination of pathogenic mutations, several different principles are at the base of the available methods, which is the reason why different results can be obtained for the same query. A single query system is under development (http://bioinfo.uta.fi/PON-P) and a method to assess and integrate the results would be welcome as well, as careful choice and understanding of the methods and their limitations is still important to avoid overprediction. At the moment, as these methods cannot find a clear correlation with a disease phenotype, specifically designed experiments are still required.

Because of all these limitations, it would be risky to consider our findings conclusive. In contrast, we believe that they can give an initial but solid structural interpretation of how mutational alterations of ALK1_EC_ can lead to HHT2, and hence a valuable framework to systematically tackle the molecular basis of its pathogenesis by biological methods.

## Materials and Methods

### Comparative protein structure modelling

The amino acid sequence of human ALK1_EC_ (residues 22–118) was taken from Uniprot entry P37023. Pcons metaserver [29]–[31] was used for identification of the three-dimensional fold. Four initial models were generated by Pcons [29]–[31], Genesilico [35], I-Tasser [37] and RaptorX [38], respectively. A fifth and final model was then obtained by running Modeller [75] using these four models as templates and secondary structure elements predicted by PSIPRED [41]. Models and structures were assessed by the Qmean server [76], the best performing and publicly available model quality assessment software in CASP9 [26], and by RAMPAGE [42], ProSA-web [43], VERIFY3D [45], ProQ [46].

### Missense mutation analysis

Twenty-nine missense mutations located in ALK1_EC,_ both deposited in the HHT database [18] and/or described by our group [19], were analysed. All modelled mutations were therefore found in HHT patients diagnosed as surely affected as reported by Shovlin *et al.* [77]. The method of Thusberg and Vihinen [22] was applied to study the effect of mutations, with the modifications and implementations described below. A total of 27 sequence homologues for ALK1_EC_ domain and sequence alignments were from the Pfam database [51]. Alignments were calculated with Mcoffee [78], MAFFT [79], Promals [80], Clustalw [81] and Muscle [82], and visualized using MultiDisp [52] and ConSeq [53] for illustration of conserved amino acids in the sequence. The default parameters were applied in all methods.

The evolutionary conservation of the sequences was studied, in addition to the visualization programs, by ProCon, a program for calculating mutual information and entropy in amino acid sequences [54]. Conservation indices were calculated with the ConSurf server [53].

Structural disorder in the protein and the effects of mutations were studied using seven methods, Disopred [83], IUPred [84], PrDOS [85] Ronn [86], Pondr [87], Poodle-S [88], Spritz [89]. The effects of mutations on aggregation propensities were studied by TANGO [90], PASTA [91], Waltz [92], AGGRESCAN [93].

The pathogenic effects of point mutations were analyzed using SIFT [94], PolyPhen [95], Pmut [96], and PhD-SNP [97]. The effects of mutations on protein stability were predicted by Scpred [60], Scide [59], Sride [61], PoPMuSiC [98], FoldX [99], Dmutant [100], Cupsat [101], Imutant [102], Mupro [103], Iptree-STAB [104] and Eris [105]. Instead of modelling the mutations manually as described by Thusberg and Vihinen [22], the BuildModel option of FoldX version 3.0 beta, whose force field is detailed in [99], was used. The BuildModel command reads the PDB and duplicates it internally. Then, it mutates the selected position in one molecule to itself and, in the other, to the variant selected, while moving the neighbouring side chains. The moving side chains and the rotamer set for them are the same in both cases, such that artefactual changes in energy due to the release, for example, of a clash in a neighbouring side chain in the mutant are prevented. The effect of the mutation is then computed by subtracting the energy of the self-mutated wild-type from that of the mutant, obtaining ΔΔG values that are provided in kilocalories per mole of ALK1_EC_ [99].

Amino acid contact analysis was performed with Sting [64] and PyMol [106]. By analyzing the wild-type protein, we could determine structurally important amino acids, which contribute to the stability of the protein, or amino acids with strong contacts that may be important for functional specificity. The analysis of changes in the contact energies for mutant structures provided hypotheses for the roles of the mutated amino acids. Electrostatic surface potentials were calculated and visualised with the PyMOL program [106] using the absolute electrostatic potential in a vacuum. Accessible surface area was calculated with Areimol [63].

### Docking

Docking analysis was performed by ClusPro [72]. In this software, a rigid body docking is performed, using ZDOCK [107] based on the fast Fourier transform correlation techniques. ZDOCK uses a scoring function based on shape complementarities, electrostatic potentials, and desolvation terms. Second, filtering is performed using empirical free energy functions and pairwise root mean square deviation clustering. The ligand with the most neighbours is the cluster center, which is then minimized by the CHARMM algorithm in the presence of the receptor. For ALK1_EC_, the homology model we generated was used, and, for BMP9 structure, the dimeric form of BMP9 (PDB ID: 1zkz) was docked.

### Visualisation of models and interface analysis

Visual inspection of the models and structures, and preparation of the figures was carried out using the program Pymol [106]. Interface analysis was performed with PISA [108].

## Supporting Information

Figure S1 **Local Qmean scores of ALK1_EC_ models.** Cartoon representation of (A) superposed ALK1_EC_ models generated by Pcons [29]-[31], Genesilico [35], I-Tasser [37] and RaptorX [38], (B) final model generated by MODELLER [40]. Molecules were coloured with a blue (low Qmean score) to red (high Qmean score) gradient.
(TIF)
