# Supplementary figures and images for: Bioinformatic Analysis of Pathogenic Missense Mutations of Activin Receptor Like Kinase 1 Ectodomain
Source: PLoS One. 2011 Oct 18;6(10):e26431. doi: 10.1371/journal.pone.0026431 (PMC3196573; doi:10.1371/journal.pone.0026431)

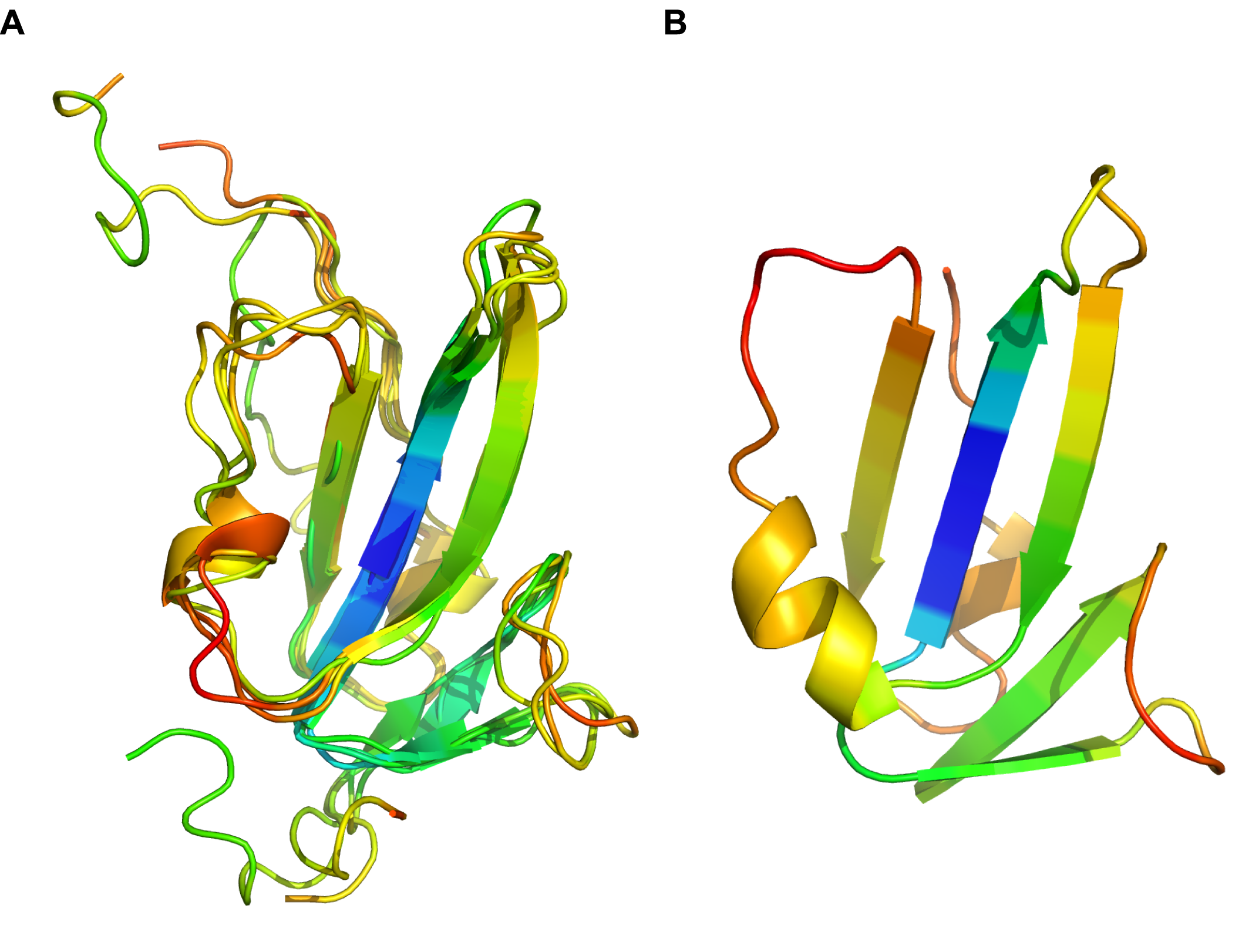

Supplement: Figure S1 — Local Qmean scores of ALK1EC models. Cartoon representation of (A) superposed ALK1EC models generated by Pcons [29]-[31], Genesilico [35], I-Tasser [37] and RaptorX [38], (B) final model generated by MODELLER [40]. Molecules were coloured with a blue (low Qmean score) to red (high Qmean score) gradient. (TIF) [file pone.0026431.s001.tif]
